# Medical Education in Diabetes Management on the New Horizon: Insights From Metabolic Management Center

**DOI:** 10.1111/1753-0407.70075

**Published:** 2025-03-19

**Authors:** Guang Ning

**Affiliations:** ^1^ Department of Endocrine and Metabolic Diseases Shanghai Institute of Endocrine and Metabolic Diseases, Ruijin Hospital, Shanghai Jiao Tong University School of Medicine Shanghai China; ^2^ National Clinical Research Center for Metabolic Diseases (Shanghai), Key Laboratory for Endocrine and Metabolic Diseases of the National Health Commission of the PR China, National Research Center for Translational Medicine, Shanghai Key Laboratory for Endocrine Tumor, State Key Laboratory of Medical Genomics Ruijin Hospital, Shanghai Jiao Tong University School of Medicine Shanghai China

## 1 Introduction

The global diabetes epidemic presents a formidable challenge to healthcare systems, with 529 million cases documented in 2021 and projections estimating a rise to more than 1.31 billion by 2050 [1]. Low‐ and middle‐income countries bear a disproportionate burden, representing 80% of global diabetes cases [2]. Notably, China has the largest population living with diabetes worldwide, with over 118 million individuals affected [1], accompanied by alarmingly low rates of awareness, treatment, and control [3, 4]. Driven by rapid urbanization, an aging population, significant environmental and lifestyle shifts, and regional and socioeconomic disparities, China faces an urgent imperative to effectively address these challenges.

The National Metabolic Management Center (MMC), launched in 2016, emerged as a response to these challenges. Guided by the principle of “One Center, One Stop, One Standard,” MMC integrates cutting‐edge medical equipment, evidence‐based protocols and multidisciplinary collaboration to redefine diabetes management [5]. The MMC establishes a nationwide network of metabolic centers across hospitals and primary healthcare facilities in China to enhance guideline‐based diabetes management. This editorial delineates MMC's development journey, emphasizing its innovations in patient care, medical education, artificial intelligence (AI)‐driven precision medicine, and global contributions to metabolic health governance.

## 2 MMC: A Paradigm Shift in Diabetes Management

MMC's operational model revolutionizes traditional fragmented care by consolidating services into a unified center. Guided by the MMC Experts Committee, a series of standard operating procedures (SOPs) and MMC‐specific guidelines have been developed to standardize the disease management. At every MMC, patients can receive one‐stop care encompassing the complete spectrum of healthcare services from initial registration, diagnostic testing, clinical assessment, therapeutic prescription, to formulation of personalized follow‐up strategies [6, 7, 8]. To ensure the nationwide implementation of this model, the MMC has established a four‐tiered prevention and control network, comprising the MMC leading center, regional centers, county centers, and community centers. A teleconsultation and referral system for complex cases has been integrated into this network, enabling seamless communication and patient transfers across different levels of MMCs. Furthermore, the MMC leverages a health information platform based on Internet of Things (IoT) and advanced technologies to support continuous and personalized care delivery, effectively bridging the gap between hospital‐based and community‐based management. Central to the MMC platform are two interconnected systems: the MMC digital medical record system and the online education system.

The digital medical record system integrates comprehensive clinical phenotyping, detailed biochemical profiling, and diabetes‐related complication indicators to create a real‐time, multidimensional data fusion framework. Leveraging advanced AI technologies, it converts laboratory test report images into editable text, automatically extracts key data, and covers over 1000 metabolic parameters, ensuring seamless data interoperability across in‐ and out‐of‐hospital settings through IoT‐enabled interfaces, including proprietary mobile applications, WeChat‐based platforms, and telemedicine systems. Complementing this, MMC has developed a comprehensive online education system featuring patient education modules and clinical training programs for healthcare providers. Together, these initiatives tackle systemic challenges in China's metabolic disease management by eliminating care fragmentation through standardized protocols, improving access to specialists via telemedicine and tiered networks, ensuring adherence to clinical guidelines through continuous medical education, and expanding the capacity for diabetes education through online training programs.

## 3 Health Education for Patients

### 3.1 In‐Hospital Health Education for Patients

In‐hospital education for patients with diabetes can be delivered through multiple approaches at every MMC. During each follow‐up visit, healthcare providers can offer personalized lifestyle interventions based on the patients' glycemic control status. Additionally, group education can be implemented, such as scheduled large classroom sessions, small group discussions, and one‐on‐one counseling.

### 3.2 Out‐Of‐Hospital Online Education for Patients

A series of applications and mini programs supported by the health information platform have been developed to provide multifaceted health education for patients: (1) Health literacy enhancement through educational materials (articles, short videos) authored by endocrinology specialists, with regular content updates ensuring clinical accuracy; (2) Home‐based health monitoring enabling real‐time uploads of glucose levels, blood pressure, and lifestyle data to facilitate physician‐patient data sharing and teleconsultations; (3) Self‐management empowerment via automated reminders for follow‐up notifications, medication adherence, dietary control, and physical activity schedules.

## 4 Medical Education for Healthcare Providers

### 4.1 Online Medical Education

Online medical education for healthcare providers constitutes an integral element of the MMC educational framework. The effective implementation of the MMC program hinges on standardized clinical training, with its curriculum strategically structured around four evidence‐based pillars in line with current guidelines: (1) diagnostic standardization: diagnostic criteria for diabetes and related metabolic disorders, emphasizing guideline‐recommended biochemical assessments and clinical evaluation protocols; (2) complication screening: standardized workflows for multi‐organ assessments, including diabetic retinopathy (DR), nephropathy, neuropathy, and cardiovascular risk evaluation; (3) therapeutic optimization: evidence‐based treatment regimens covering lifestyle modification and medication intervention strategies; (4) MMC operational competency: patient management workflows, medical history and biological specimen collection, and proficiency in MMC digital platforms. Furthermore, the online curriculum incorporates lectures by experts in the field of endocrine and metabolic disorders, covering SOP training, guideline interpretation, expert experience sharing, and academic article writing. Through these structured educational modules, healthcare providers can achieve an in‐depth understanding of diabetes management principles and MMC's operational workflows.

### 4.2 Online Training Portals for Healthcare Providers

The MMC online education platform provides both web‐ and application‐based training portals, facilitating easy access to educational resources and competency assessments for healthcare providers. Certification courses are predominantly delivered through the MMC Healthcare Workstation or its application. Currently, the number of healthcare providers registered on the MMC online education platform has reached 11 000. In addition, Global‐metab.com, an academic website specializing in endocrine and metabolic disorders, offers professional information and educational training resources for healthcare providers. Utilizing a stratified education approach, this website provides a structured and systematic framework tailored to varying levels of expertise and professional backgrounds.

### 4.3 Certification of Healthcare Providers

Before operationalizing MMC services, all healthcare providers must successfully complete a comprehensive competency evaluation and certification process to ensure adherence to standardized care protocols. To obtain this certification, physicians and nurses must meet the following requirements: (1) completing mandatory online training modules; (2) achieving a minimum passing score of 90% on an online examination, which is derived from a dynamic question bank and systematically evaluates compliance with SOPs across the entire MMC “one‐stop” workflow; (3) demonstrating proficiency in MMC operations through on‐site assessments. The electronic certification credential is integrated into the MMC education system, facilitating real‐time tracking of competency.

### 4.4 Exploring Diversified Medical Educational Models Integrating Online and Offline Approaches

While digital‐enabled online training has enhanced the accessibility and flexibility of medical education, emerging evidence underscores the critical need for MMC to pioneer an integrated online‐offline medical educational framework that bridges competency‐based clinical decision‐making with real‐world patient management challenges. The Chinese Endocrinologists Health Education Study (CREATION) exemplifies such an endeavor within the MMC platform (ClinicalTrials.gov NCT05715307). This multicenter cluster‐randomized trial is designed to investigate whether a physician‐centered, role‐playing‐based intensive training program, combined with the MMC's existing online education infrastructure could enhance physicians' practical competencies and improving metabolic goal achievement among their cared patients with type 2 diabetes. This hybrid educational paradigm is anticipated to establish a virtuous cycle of “knowledge transfer—skill mastery—behavioral transformation—care quality enhancement,” thereby driving sustainable quality improvement in metabolic disease management.

## 5 Quality Accreditation of MMC Management

To ensure real‐time supervision and quality certification of MMC workflows and management standards, the MMC Experts Committee has established standardized monitoring processes and qualification systems.

### 5.1 MMC Centers Accreditation

MMC facilities demonstrating compliance with predefined operational standards may initiate accreditation applications through the Quality Control (QC) team. The certification protocol encompasses rigorous evaluation of standardized clinical workflows and data integrity metrics (including completeness, accuracy, and timeliness). Details regarding QC measures can be found on the ClinicalTrials.gov website (NCT03811470). Formal accreditation is conferred via an official endorsement ceremony. Post‐accreditation continuous quality monitoring include: real‐time performance dashboards quality indicators tracking, regular manual QC, and annual quality reviews.

### 5.2 Annual Comprehensive Management Ranking

The MMC network conducts annual comprehensive assessments and ranking of every center through a multidimensional scoring system across five critical domains: data quality (including patient volume, follow‐up rates, data completeness, and sample collection), clinical outcomes (measured by metabolic control rates and complication screening rates), health screening initiatives, multidisciplinary team development, and hierarchical network integration. This system categorizes the management quality of MMCs into three distinct tiers: low, medium, and high. This systematic evaluation mechanism has proven effective in driving substantial enhancements in care quality, supported by a robust framework of regular performance feedback, tailored capacity‐building initiatives for underperforming centers, and annual best practice exchange forums.

## 6 AI‐Enabled Digital Tools

The MMC initiative has pioneered the integration of AI technologies into diabetes management through three transformative applications.

### 6.1 Risk Prediction Models

*Rui Ning Zhi Tang*: A predictive tool estimating 3‐year diabetes risk with personalized prevention strategies, enhancing early detection and targeted intervention.

*Rui Ning Yu Tang*: This predictive algorithm, referred to as the “metabolic index,” employs six conventional clinical parameters to estimate cardiovascular risk profiles within a 3‐year prognostic timeframe. This model enables the early identification of high‐risk patients for cardiovascular complications.

### 6.2 AI‐Enhanced DR Screening

A cohort of nearly 50 000 diabetic patients from selected MMC DR centers underwent comprehensive DR screening through automated deep learning (DL) algorithm‐based analysis of fundus photographs [9]. This AI solution demonstrated a sensitivity of 83.3% and specificity of 92.5% in identifying referable DR cases. It has significantly reduced screening time while maintaining diagnostic accuracy, validated by ophthalmology specialists.

### 6.3 Intelligent Decision‐Making

*Rui Ning Zhu Tang*, also called the “Dia‐Master,” revolutionizes precision medication recommendation through a sophisticated three‐tiered architecture, enabling AI‐driven, patient‐specific drug therapy optimization that integrates individual clinical profiles with evidence‐based guidelines. This approach bridges the gap between personalized care and standardized protocols, ensuring tailored medication decisions that align with each patient's unique therapeutic needs while maintaining adherence to best practices.

## 7 Current Progress and Future Perspectives

The MMC network has expanded significantly, encompassing more than 2000 metabolic centers across 32 provinces, autonomous regions, and municipalities in China, and has provided care to 3 million patients with diabetes nationwide. Empowered by a proprietary health information platform supporting dynamic big data analysis and AI technology‐based medical tools, the MMC network would continue to grow. Preliminary data show that under MMC management, the proportion of achieving glycated hemoglobin (HbA1c) < 7.0% in patients with diabetes surged from 22.7% to 53.2%. The rates of patients who met all three treatment targets (HbA1c < 7%, blood pressure < 140/90 mmHg, and low density lipoprotein‐cholesterol < 2.6 mmol/L) nearly tripled from 7.3% to 19.8%. MMC's decade‐long evolution demonstrates that standardization, digitalization, and medical education can dismantle barriers to equitable diabetes care.

By enabling global data sharing and fostering international collaboration, the MMC platform accelerates large‐scale, real‐world evidence generation while establishing a globally referential model for medical education. This integrated approach yields critical insights that advance our understanding of diabetes pathophysiology and treatment optimization, while simultaneously revolutionizing diabetes care training through standardized, data‐driven educational frameworks that are being adopted worldwide.

## Conflicts of Interest

The author declares no conflicts of interest.
